# Responses to incremental exercise and the impact of the coexistence of HF and COPD on exercise capacity: a follow-up study

**DOI:** 10.1038/s41598-022-05503-5

**Published:** 2022-01-31

**Authors:** Polliana B. Dos Santos, Rodrigo P. Simões, Cássia L. Goulart, Guilherme Peixoto Tinoco Arêas, Renan S. Marinho, Patrícia F. Camargo, Meliza G. Roscani, Renata F. Arbex, Claudio R. Oliveira, Renata G. Mendes, Ross Arena, Audrey Borghi-Silva

**Affiliations:** 1 Cardiopulmonary Physical Therapy Laboratory, Federal University of São Carlos - UFSCar, Sao Carlos, São Paulo Brazil; 2 Sciences of Motricity Institute, Postgraduate Program in Rehabilitation Sciences, Federal University of Alfenas, Alfenas, MG Brazil; 3 Department of Physiological Sciences, Federal University of Amazonas, Manaus, Brazil; 4 Department of Medicine, Federal University of Sao Carlos, Sao Carlos, Brazil; 5 Department of Physical Therapy, College of Applied Health Sciences, University of Illinois at Chicago, Chicago, IL USA

**Keywords:** Cardiology, Diseases

## Abstract

Our aim was to evaluate: (1) the prevalence of coexistence of heart failure (HF) and chronic obstructive pulmonary disease (COPD) in the studied patients; (2) the impact of HF + COPD on exercise performance and contrasting exercise responses in patients with only a diagnosis of HF or COPD; and (3) the relationship between clinical characteristics and measures of cardiorespiratory fitness; (4) verify the occurrence of cardiopulmonary events in the follow-up period of up to 24 months years. The current study included 124 patients (HF: 46, COPD: 53 and HF + COPD: 25) that performed advanced pulmonary function tests, echocardiography, analysis of body composition by bioimpedance and symptom-limited incremental cardiopulmonary exercise testing (CPET) on a cycle ergometer. Key CPET variables were calculated for all patients as previously described. The ${\dot{\text{V}}}$_E_/${\dot{\text{V}}}$CO_2_ slope was obtained through linear regression analysis. Additionally, the linear relationship between oxygen uptake and the log transformation of ${\dot{\text{V}}}$_E_ (OUES) was calculated using the following equation: ${\dot{\text{V}}}$O_2_ = a log ${\dot{\text{V}}}$_E_ + b, with the constant ‘a’ referring to the rate of increase of ${\dot{\text{V}}}$O_2_. Circulatory power (CP) was obtained through the product of peak ${\dot{\text{V}}}$O_2_ and peak systolic blood pressure and Ventilatory Power (VP) was calculated by dividing peak systolic blood pressure by the ${\dot{\text{V}}}$_E_/${\dot{\text{V}}}$CO_2_ slope. After the CPET, all patients were contacted by telephone every 6 months (6, 12, 18, 24) and questioned about exacerbations, hospitalizations for cardiopulmonary causes and death. We found a 20% prevalence of HF + COPD overlap in the studied patients. The COPD and HF + COPD groups were older (HF: 60 ± 8, COPD: 65 ± 7, HF + COPD: 68 ± 7). In relation to cardiac function, as expected, patients with COPD presented preserved ejection fraction (HF: 40 ± 7, COPD: 70 ± 8, HF + COPD: 38 ± 8) while in the HF and HF + COPD demonstrated similar levels of systolic dysfunction. The COPD and HF + COPD patients showed evidence of an obstructive ventilatory disorder confirmed by the value of %FEV_1_ (HF: 84 ± 20, COPD: 54 ± 21, HF + COPD: 65 ± 25). Patients with HF + COPD demonstrated a lower work rate (WR), peak oxygen uptake (${\dot{\text{V}}}$O_2_), rate pressure product (RPP), CP and VP compared to those only diagnosed with HF and COPD. In addition, significant correlations were observed between lean mass and peak ${\dot{\text{V}}}$O_2_ (r: 0.56 p < 0.001), OUES (r: 0.42 p < 0.001), and O_2_ pulse (r: 0.58 p < 0.001), lung diffusing factor for carbon monoxide (D_LCO_) and WR (r: 0.51 p < 0.001), D_LCO_ and VP (r: 0.40 p: 0.002), forced expiratory volume in first second (FEV_1_) and peak ${\dot{\text{V}}}$O_2_ (r: 0.52; p < 0.001), and FEV_1_ and WR (r: 0.62; p < 0.001). There were no significant differences in the occurrence of events and deaths contrasting both groups. The coexistence of HF + COPD induces greater impairment on exercise performance when compared to patients without overlapping diseases, however the overlap of the two diseases did not increase the probability of the occurrence of cardiopulmonary events and deaths when compared to groups with isolated diseases in the period studied. CPET provides important information to guide effective strategies for these patients with the goal of improving exercise performance and functional capacity. Moreover, given our findings related to pulmonary function, body composition and exercise responses, evidenced that the lean mass, FEV_1_ and D_LCO_ influence important responses to exercise.

## Introduction

The incidence and prevalence of chronic-degenerative diseases in a progressively elderly population has increased worldwide over the last several decades^1^. In this context, cardiopulmonary disease (CPD) is increasing and remains the leading cause of death in many countries; in Brazil, CPD is responsible for approximately 20% of all deaths in adults 30 years of age or older^2,3^.

Heart failure (HF) and chronic obstructive pulmonary disease (COPD) are predominant chronic diseases; the growing prevalence of HF and COPD reflects a combination of aging population, increasing incidence of both diseases in association with diagnostic access^4–6^. The pathophysiology of HF and COPD are well established in the literature^7^. HF is a syndrome initially characterized by cardiac dysfunction resulting from structural changes or cardiac function, however more advanced stages of the disease leads to compromised lung function and systemic changes, such us pulmonary restrictions, ventilation-perfusion abnormalities, pulmonary congestion and reduced peripheral muscle mass with consequent onset of fatigue^7–9^.

On the other hand, COPD is a common, preventable, and treatable disease characterized by persistent respiratory symptoms and airflow obstruction^10^. The inflammation resulting from exposure to COPD triggering factors causes a cascade of events that generate pathological abnormalities such as changes in cardiac structure and function and muscle atrophy leading to the onset of dyspnea and inactivity with a consequent reduction in functional capacity^11–13^.

HF and COPD share similar signs and symptoms, and often coexist, leading to a worse prognosis, as well as greater challenges for diagnosis and the establishment of therapeutic interventions. It is estimated that the prevalence of HF in patients with COPD and vice versa is between 10 and 25% in developed countries^11,14^. The overlap of HF and COPD is associated with increased morbidity, poorer quality of life and greater use of health resources^15^.

One of the most common and impactful symptoms in patients with either independent or overlapping HF and COPD is decreased exercise capacity; a commonly reported subjective symptom in dyspnea with exertion^16,17^.

In patients with overlap where HF prevails, there is a loss in the delivery or use of oxygen (O_2_). This is because the stroke volume reduction resulting from cardiac dysfunction is not able to compensate for the high muscle O_2_ extraction, leading to a low delivery for muscle contraction, causing the anaerobic metabolism to happen earlier, thus increasing lactate rates circulating and thus causing an early sensation of muscle fatigue^18^. In addition, ventilatory changes also play an important role in exercise limitation: excessive ventilatory stimulation resulting from high cardiac filling pressures increases reflex chemosensitivity, increasing ventilation in relation to demands, moreover, restrictive changes in ventilatory mechanics also occur, increasing thus the feeling of dyspnea on exertion^19^.

In overlap patients, where COPD has a greater influence on exercise limitation, ventilatory inefficiency is evidenced. The Inflammation, fibrosis, and exudates in the small airways of patients with COPD generate a reduction in FEV1 and progressive airway obstruction during expiration, resulting in lung hyperinflation, reduced inspiratory capacity that influences the increase in functional residual capacity (FRC), resulting in increased dyspnea, which associated with a reduction in the intrinsic contractile properties of the ventilatory muscles and abnormalities in ventilation perfusion, generates ventilatory inefficiency and limited exercise capacity^20,21^.

A detailed assessment of exercise capacity is relevant in these patient populations from diagnostic, prognostic and therapeutic efficacy perspective. Cardiopulmonary exercise testing (CPET) is the gold-standard approach to assessing exercise capacity and more broadly cardiorespiratory fitness; an evidence-based panel of core CPET measures allows for a more comprehensive evaluation^22^. Through CPET, ventilatory and gas exchange, as well as heart rate (HR), electrocardiogram, and blood pressures, are measured to provide detailed information on the cardiovascular, pulmonary, and muscular systems as well as detect the primary limitation to exercise in these chronic conditions^23^.

The coexistence of HF and COPD has important therapeutic and prognostic implications, the knowledge about the prevalence of the concomitance of these diseases is clinically relevant. Moreover, follow-up studies have contributed substantially to the understanding of disease progression, clinical outcomes, mortality, and use of resource in health, as both conditions are quite disabling, generating heightened concern when these conditions coexist.

In the present study, we hypothesized that HF + COPD would further deteriorate cardiorespiratory fitness compared to patients diagnosed with HF and COPD in isolation. We additionally hypothesized that there are relationships between clinical characteristics and CPET measures of cardiorespiratory fitness. The specific aims of this study was to assess: (1) the prevalence of HF and COPD overlap in the studied patients; (2) the impact of the overlapping HF + COPD on exercise capacity and cardiorespiratory fitness and to contrast these measures in patients with either HF or COPD in isolation; and (3) the relationship between clinical characteristics and measures of cardiorespiratory fitness (4) verify the occurrence of exacerbations, hospitalizations and deaths in the follow-up period of up to 24 months.

## Methods

### Study design and subjects

This longitudinal study was reported following recommendations of the Strengthening the Reporting of Observational Studies in Epidemiology (STROBE) statement^24^. Three-hundred fifteen patients were screened from 3 cardiology and pneumology outpatient clinics of the University Hospital of the Federal University of São Carlos, from 01 June 2017 to 30 December 2019. All patients who attended during this period with the diagnosis of HF with reduced or borderline ejection fraction (EF) and/or COPD were contacted by phone and was asked questions regarding diagnosis, clinical conditions, disease stability, drug optimization, and functional mobility. For all patients, eligibility criteria were: (1) age range of 40–85 years; (2) clinically stable for at least 3 months (i.e., no worsening of symptoms, exacerbation or decompensation); (3) no change in dose or change in medication for at least 3 months; (4) no hospitalizations for any cause for at least 3 months; and (5) absence of any condition that may affect exercise performance (e.g., anemia, neuromuscular disorders, or malignancies). Non-inclusion criteria were: (1) long-term O_2_ therapy; (2) musculoskeletal disease that would impact exercise performance (e.g., osteoarthritis, osteonecrosis, trauma, etc.); and (3) peripheral arterial disease associated with claudication. Moreover, HF or COPD exacerbation or hospitalization during the study was a criterion for study drop-out. All patients who met the eligibility criteria were invited for an initial assessment and tests to confirm the diagnosis of one (HF or COPD) or both (HF + COPD) diseases being assessed in the current study.

**Figure 1:**
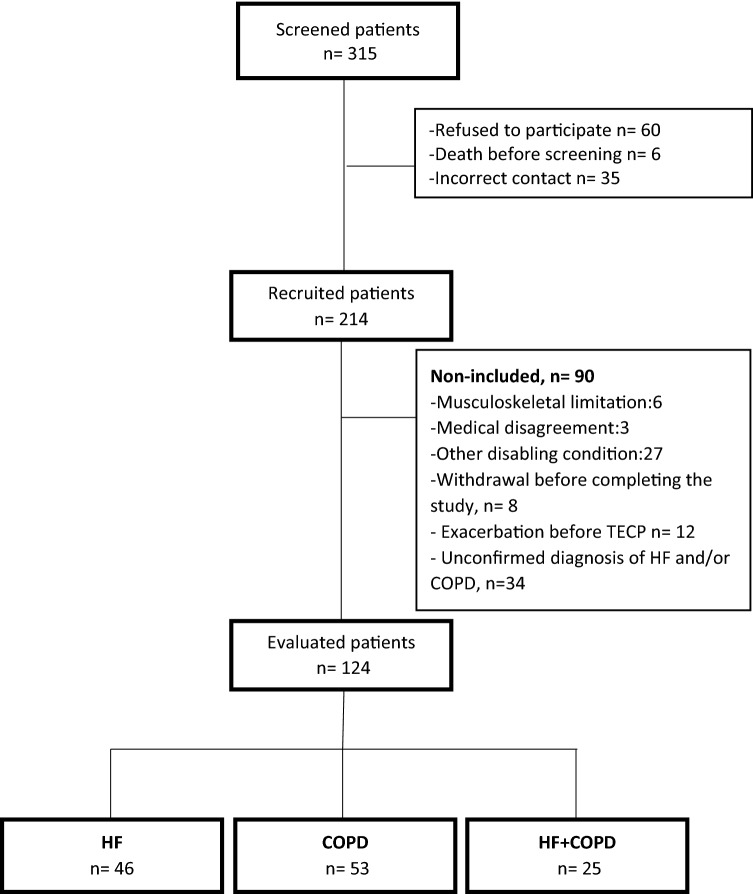
Study flow chart.

Disease treatment was optimized before study entry and patients underwent CPET only after an agreement had been reached between pneumologists and cardiologists regarding disease stability. As showed in Fig. 1, 124 patients with a confirmed diagnosis of HF and/or COPD were included. The Study followed the resolution no. 466 of the National Health Council (current guideline in Brazil) and The Declaration of Helsinki and was approved by the Ethics and Research Committee of the Federal University of São Carlos. All participants were informed about the objectives, experimental procedures and potential risks involved in this study and gave written informed consent statement prior to participation.

### Cardiac and lung function assessments

All patients underwent a transthoracic two-dimensional and Doppler echocardiographic (HD11 XE, Philips, Amsterdam, Netherlands) examination at baseline to confirm the diagnosis, stratify the degree of systolic dysfunction, and obtain the necessary measures for cardiac function in the HF and HF + COPD groups and confirm the absence of reduced ejection fraction in the COPD group. Patients with HF were determined according to left ventricle ejection fraction ≤ 50%^25^. Advanced pulmonary function assessment (Masterscreen Body, Mijnhardt/Jäger, Würzburg, German) was performed to obtain dynamic and static lung volumes and capacities, such us Forced Expiratory Volume in 1 s (FEV_1_), Forced Vital Capacity (FVC), Residual Volume (RV), Total Lung Capacity (TLC), Inspiratory Capacity (IC), and Diffusion Capacity Carbon Monoxide (D_LCO_) pre and post-bronchodilator therapy. The interruption of the use of bronchodilators was requested 12 h before the exam and the GOLD criteria [post-bronchodilator FEV_1_/ FVC ratio < 0.70] was used to confirm a COPD diagnosis^26^.

### Cardiopulmonary exercise testing

All patients underwent a symptom-limited CPET on an electronically braked cycle ergometer (Corival Recumbent, Lode, Groningen, Netherlands) using the Oxycon Mobile System (Mijnhardt/Jäger, Würzburg, German). The patients’ continuous use medications were kept for the test. The exercise protocol started with 5 min of data collection at rest, followed by unloaded cycling for 1 min with a subsequent increment of 5–10 watts each minute (ramp protocol). Patients were instructed to pedal at the cadence of 60 rotation per minute and the work rate (WR) increment was individually selected according to reported exercise tolerance. Breath-by-breath ${\dot{\text{V}}}$O_2_ (L/min), ${\dot{\text{V}}}$CO_2_ (L/min), and ${\dot{\text{V}}}$_E_ (L/min) were recorded. The CPET variables were reported as 20-s averaged data. During the rest, exercise test and the recovery phase, the HR (collected with heart rate monitor), 12-lead electrocardiogram (ECG), blood pressure (collected through manual auscultation), and arterial oxygen saturation were monitored. Arterial oxygen saturation was measured non-invasively by pulse oximetry (SpO_2_, %). Breathlessness and leg effort scores were rated according to the 10-point Borg category ratio scale^27^. Established criteria to interrupt the test were followed and included angina (score above 2 on a scale of 0–10), life-threatening arrhythmias, electrocardiographic evidence of ischemia, a drop in systolic blood pressure, or arterial oxygen saturation ≤ 84%^28^. Key CPET variables were calculated for all patients as previously described. The ${\dot{\text{V}}}$_E_/${\dot{\text{V}}}$CO_2_ slope was obtained through linear regression analysis^29^. Additionally, the linear relationship between oxygen uptake and the log transformation of ${\dot{\text{V}}}$_E_ (OUES) was calculated using the following equation: ${\dot{\text{V}}}$O_2_ = a log ${\dot{\text{V}}}$_E_ + b, with the constant ‘a’ referring to the rate of increase of ${\dot{\text{V}}}$O_2_^30^. Circulatory power (CP) was obtained through the product of peak ${\dot{\text{V}}}$O_2_ and peak systolic blood pressure and Ventilatory Power (VP) was calculated by dividing peak systolic blood pressure by the ${\dot{\text{V}}}$_E_/${\dot{\text{V}}}$CO_2_ slope^31,32^.

### Patients follow-up

All patients were followed for 24 months. The follow-up of patients started after the performance of the CPET and was carried out through telephone contact every 6 months (6, 12, 18, 24 months), where the patient or caregiver (in case the patient was unable to respond) answered a questionnaire with 5 questions regarding occurrence of cardiopulmonary events such as diseases exacerbations, hospitalizations by cardiopulmonary causes [acute myocardial infarction (AMI), stroke, cardiac or pulmonary surgery] and death.

### Statistical analysis

A sample calculation was performed (GPower 3.1—University of Kiel, Kiel, Germany) using the peak ${\dot{\text{V}}}$O_2_ (HF: 12 ± 3, COPD: 14 ± 3, HF + COPD: 11 ± 3; *p*: 0, 17; power: 0.80) obtained in pilot studies previously performed in our laboratory with individuals who were diagnosed with HF and COPD. From this sample calculation, 42 subjects, 14 for each group, were needed to reach sufficient statistical power of 0.80. The Shapiro–Wilk test was used to verify the data distribution. Descriptive variables were expressed as mean ± standard deviation, when normal distribution was present, or median and interquartile, when non-normal distribution was not present. Categorical variables are expressed as frequencies and percentages and compared using the chi-square test. A one-way ANOVA was used to compare anthropometric measures, cardiac and pulmonary function measures and CPET measures. A two-way ANOVA was used to compare the exercises responses between groups at different times of exercise. Relationships between measures collected in the current study were assessed by the Pearson Correlation coefficient which correlation strengths will be classified as trivial— < 0.1, small—0.30–0.50, large—0.50–0.70, very large— > 0.70–0.90, nearly perfect— > 0.90^33^. The analysis of the occurrence of events: number of disease exacerbations, number of hospitalizations for cardiopulmonary causes in the period, onset of AMI, stroke, cardiac or pulmonary surgery and death from cardiopulmonary causes was evaluated by the analysis of Klapan-Meier survival with the groups being compared using the Log-rank test. A p-value < 0.05 was considered as statistically significant for all tests. All statistical analyses were performed using the Statistical Package for the Social Sciences (SPSS) 20.0 (IBM, Armonk, New York) and PRISM 9.0 (GraphPad, San Diego, California).

## Results

### Clinical and resting characteristics

**Figure 2:**
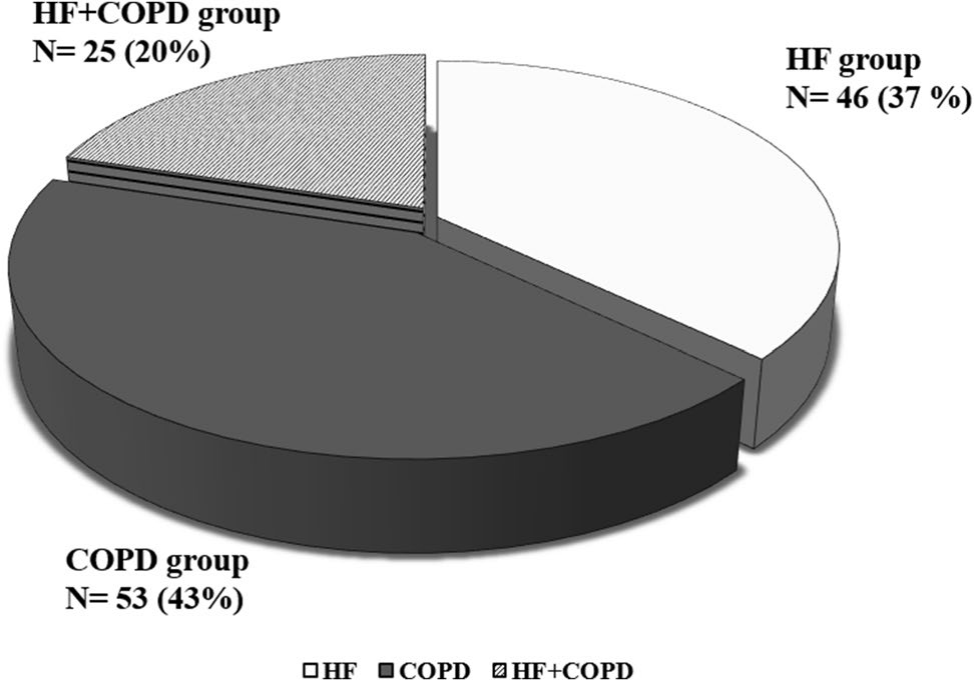
Prevalence of patients with HF + COPD overlap in studied populations. *HF* heart failure, *COPD* chronic obstructive pulmonary disease.

Figure 2 demonstrates that the prevalence of coexisting HF and COPD diagnoses in the studied patients was 20%. Of the 25 patients who were included in the HF + COPD group, only 18 patients (72%) had a previous diagnosis of overlap, while 7 (28%) patients were diagnosed with the overlap after performing the echocardiogram or advanced pulmonary assessment in our study. The 7 patients where a diagnosis of HF or COPD was newly identified were referred for medication optimization and, after 3 months, returned to complete data collection for the current study.

Patient characteristics are reported in Table 1. Significant differences were found for most anthropometric and clinical variables amongst HF, COPD and HF + COPD groups. Most patients in both groups were male while patients in the COPD and HF + COPD groups were older (p < 0.05). In relation to cardiac function, as expected, patients with COPD presented with preserved systolic function, while in the HF and HF + COPD demonstrated similar levels of systolic dysfunction, although differences between HF and HF + COPD groups were found in systolic dysfunction staging (p < 0.05). Differences were found in the Mitral E wave in the HF and COPD groups. As expected, COPD and HF + COPD patients showed evidence of an obstructive ventilatory disorder. The frequency of patients in stage 2 according to the GOLD guidelines was greater in the COPD group. HF patients showed lower static lung volumes when compared to the COPD group (p < 0.05); differences were also found in Residual volume (RV), % predicted TLC and D_LCO_. In relation to the presence of comorbidities, the HF group was the most affected when contrasted with the COPD group.

**Table 1:** Anthropometric and clinical characteristics of studied subjects.

Variables	HF n = 46	COPD n = 53	HF + COPD n = 25	*p*
Age, years	60 ± 8	65 ± 7*	68 ± 7*	< 0.000
Gender, M/F (n)	32/14	37/16	25/0	0.007
Height, m	1.66 ± 0.93	1.66 ± 0.26	1.75 ± 0.1*^#^	< 0.000
Weight, kg	80 ± 16	69 ± 15*	71 ± 14	0.003
BMI, kg/m^2^	29 ± 6	25 ± 4*	25 ± 4*	0.002
Lean mass, %	50 ± 10	42 ± 8*	48 ± 9	0.02
Fat-free mass, %	53 ± 10	45 ± 8*	51 ± 10	0.01
Body fat, %	26 ± 9	20 ± 9	19 ± 8	0.06
Protein, %	10.4 ± 2	8 ± 1*	10.1 ± 2	0.02
Minerals, %	3.6 ± 0.7	2.9 ± 0.5*	3.4 ± 0.7	0.005
**Cardiac function**				
Ejection fraction, %	40 ± 7	70 ± 8*	38 ± 8^#^	< 0.000
Mild/moderate/severe LV dysfunction (n)	26/17/3	–	10/11/4	< 0.000
Indexed LA volume, mL/m^2^	39 ± 18	36 ± 13	44 ± 11	0.21
Mitral E wave, cm/s	76 ± 24	63 ± 14*	68 ± 25	0.03
Mitral e’ wave, cm/s	7 ± 2	9 ± 2	7 ± 5	0.30
E/e’ ratio, cm/s	11 ± 7	8 ± 3	10 ± 6	0.11
RVD, mm	33 (30–44)	32 (25–37)	33 (31–41)	0.14
**Pulmonary function**				
FEV_1_, L/s	2.55 ± 0.7	1.38 ± 0.9	2.00 ± 0.6	0.49
FEV_1_, % predicted	84 ± 20	54 ± 21*	65 ± 25*	< 0.000
FVC, L/s	3.31 ± 0.9	2.80 ± 2	3.40 ± 0.8	0.65
FVC, % predicted	89 ± 16	79 ± 25	87 ± 22	0.05
FEV_1_/FVC, L/s	0.78 ± 0.7	0.52 ± 0.1*	0.59 ± 0.1*	0.03
RV, L	2.6 ± 1.1	3.9 ± 1.7*	3.3 ± 1.0	0.004
RV, % predicted	127 ± 49	203 ± 93*	143 ± 44	< 0.000
TLC, L	5.2 ± 1.6	6.0 ± 1.7	5.9 ± 1.3	0.19
TLC, % predicted	88 ± 24	112 ± 36*	99 ± 30	0.01
RV/TLC	0.48 ± 0.12	0.65 ± 0.15*	0.55 ± 0.11	< 0.000
IC, L	1.9 ± 0.8	1.6 ± 0.8	1.3 ± 0.5	0.08
IC, % predicted	75 ± 32	70 ± 25	51 ± 19	0.07
DLCO, mL/ mim/mmHg	20 ± 5	13 ± 5*	17 ± 6	0.001
DLCO, % predicted	82 ± 13	61 ± 23*	72 ± 20	0.002
GOLD stage, I/II/III/IV	–	7/28/14/4	9/11/5/0	< 0.000
Pack/years	40 ± 33	83 ± 77*	59 ± 30	0.005
**Functional classification**				
NYHA, I/II/II/IV	20/20/6/0	–	8/11/6/0	0.70
mMRC, 0/I/II/III/IV	–	10/24/11/4/4	4/12/5/1/3	0.92
**Comorbities**				
Hypertension, n (%)	33	31	22*^#^	0.01
DM, n (%)	15^#^	2	11^#^	< 0.000
CI, n (%)	8	5*	5*	0.005
OSA, n (%)	4^#^	18	1^#^	0.001
Dyslipidemia, n(%)	19^#^	8	15^#^	< 0.000
Other, n (%)	28	23	15	0.19
Comorbities per patients, n (%)	3.2 ± 1	2.0 ± 1*	3.0 ± 1	0.001
**Medications**				
Betablockers, n	46	–	24	< 0.000
Bronchodilator, n	1^#^	53	21	< 0.000
Antihypertensive, n	38^#^	50	20^#^	< 0.000
Diuretics, n	35	40*	24	< 0.000

Used ANOVA one way for continuous variables and used chi-square test for categorical variables.

*HF* heart failure, *COPD* chronic obstructive pulmonary disease, *M* male, *F* female, *BMI* body mass index, *LV* left ventricle, *LA* left atrium, *RVD* right ventricle diameter, *FEV*_*1*_ forced expiratory volume in 1 s, *FVC* forced vital capacity, *RV* residual volume, *TLC* total lung capacity, *IC* inspiratory capacity, *DLCO* diffusion capacity carbon monoxide, *NYHA* New York Heart Association, *mMrc* modified Medical Research Council scale, *DM* diabetes mellitus, *CI* coronary insufficiency, *OSA* obstructive sleep apnea. Patients that peformed DLCO: 58 (HF:28, COPD:20, HF + COPD:10).

*Significant difference (p < 0.05) in relation to the HF group.

^#^Significant difference (p < 0.05) in relation to the COPD group.

### Metabolic, cardiovascular and ventilatory responses to exercise

Table 2 lists the responses to the CPET and the comparisons between groups. The WR and ${\dot{\text{V}}}$O_2_ at peak exercise were significantly lower in the HF + COPD group when compared to the HF group (p < 0.05). Similar ventilatory responses were found between groups; however, the COPD group had higher ${\dot{\text{V}}}$_E_/${\dot{\text{V}}}$CO_2_ intercept values compared to the other groups. Ventilatory power (PV) was significantly higher in the COPD group when compared to the HF + COPD group (p < 0.05). However, the COPD group demonstrated a significantly lower O_2_ pulse compared to the HF group (p < 0.05). The HR recovery was worse in the COPD group when compared to the HF group, and in relation to systolic and diastolic blood pressure at peak exercise, the HF + COPD group presented lower values compared to the other groups (p < 0.05).

**Table 2:** Comparison between group responses to incremental CPET.

Variables	HF n = 46	COPD N = 53	HF + COPD n = 25	*p*
WR, Watts	75 ± 32	61 ± 34	54 ± 21*	0.02
WR_predicted_, Watts	125 ± 27	111 ± 20*	107 ± 25*	0.003
WR_% of predicted_, Watts	59 ± 21	55 ± 28	51 ± 17	0.38
**Metabolic responses**				
*V̇O*_*2* predicted_, mL min	1891 ± 352	1625 ± 269*	1586 ± 296*	< 0.000
*V̇O*_*2* peak_, mL min	1011 ± 414	859 ± 228	806 ± 300*	0.02
*V̇O*_*2*% of predicted_, %	53 ± 16	53 ± 14	51 ± 15	0.81
*V̇O*_*2* peak_, mL kg^−1^ min^−1^	12.5 ± 3	12.3 ± 3	12.1 ± 3	0.88
*V̇O*_*2*_/WR, mL min W	14 ± 4	16 ± 7	15 ± 5	0.20
*V̇CO*_*2*_, mL min	1127 ± 430	908 ± 371*	732 ± 306*	0.001
RER_peak_	1.08 ± 0.1	1.04 ± 0.1	1.05 ± 0.09	0.12
**Cardiovascular responses**				
HR_rest_, bpm	72 ± 10	75 ± 10	79 ± 16	0.09
HR_maximal_, bpm	168 ± 8	154 ± 7*	151 ± 7*	< 0.000
HR_peak_, bpm	118 ± 21	120 ± 17	110 ± 25	0.13
HR_% of maximal_	73 ± 12	77 ± 12	72 ± 17	*0.25*
HR_rec_, bpm	101 ± 22	110 ± 18	102 ± 22	0.09
∆ HR_rec_, bpm	17 ± 17	8 ± 9*	13 ± 18	0.02
SBP_rest_, mmHg	122 ± 14	133 ± 14*	119 ± 22^#^	< 0.000
DBP_rest_, mmHg	79 ± 10	83 ± 10	77 ± 11^#^	0.03
SBP_peak_, mmHg	190 ± 31	198 ± 28	169 ± 43*^#^	0.003
DBP_peak_, mmHg	107 ± 15	104 ± 17	97 ± 23	0.08
Peak O_2_ pulse, mL/beat	8.5 ± 3	7.1 ± 2*	7.7 ± 3	0.05
RPP, bpm mmHg	22,238 ± 6875	24,041 ± 5730	19,090 ± 7713^#^	0.01
CP, mmHg mL kg^−1^ min^−1^	2439 ± 857	2451 ± 741	1987 ± 685^#^	0.03
**Ventilatory responses**				
${\dot{\text{V}}}$_E peak_, L min	43(35–54)	36(28–44)	36(26–51)	0.07
${\dot{\text{V}}}$_E_/VCO_2_ slope	37 ± 10	35 ± 11	38 ± 13	0.51
${\dot{\text{V}}}$_E_/VCO_2_ intercept, L/min	1.0 ± 2	3.0 ± 3*	0.7 ± 2^#^	0.001
OUES	1.3 ± 0.4	1.1 ± 0.5	1.2 ± 0.3	0.09
VP, mmHg	5.3 ± 1	5.5 ± 1	4.3 ± 1^#^	0.01
**Gas exchange responses**				
SaO_2rest_, %	96 ± 1	93 ± 2*	95 ± 2^#^	< 0.000
SaO_2peak_, %	94 ± 3	87 ± 7*	94 ± 3^#^	< 0.000
**Perception of symptoms**				
Peak dyspnea score, 0–10	4 ± 3	6 ± 2*	6 ± 2*	0.007
Peak leg effort score, 0–10	4 ± 3	3 ± 3	6 ± 3^#^	0.03

Used ANOVA one way for continuous variables and used chi-square test for categorical variables.

*HF* heart failure, *COPD* chronic obstructive pulmonary disease, *M* male, *F* female, *BMI* body mass index, *LV* left ventricle, *LA* left atrium, *RVD* right ventricle diameter, *FEV*_*1*_ forced expiratory volume in 1 s, *FVC* forced vital capacity, *RV* residual volume, *TLC* total lung capacity, *IC* inspiratory capacity, *DLCO* diffusion capacity carbon monoxide, *NYHA* New York Heart Association, *mMrc* modified Medical Research Council scale, *DM* diabetes mellitus, *CI* coronary insufficiency, *OSA* obstructive sleep apnea. Patients that peformed DLCO: 58 (HF:28, COPD:20, HF + COPD:10).

*Significant difference (p < 0.05) in relation to the HF group.

^#^Significant difference (p < 0.05) in relation to the COPD group.

The presence of hypoxemia on exertion, assessed by SpO_2_, did not differ between the HF and HF + COPD groups, but it was significantly reduced in the COPD group (p < 0.05). The subjective perception of exertion (i.e., dyspnea and fatigue in the legs) was higher in the COPD and HF + COPD groups, the main reason for the end of the test being dyspnea.

**Figure 3:**
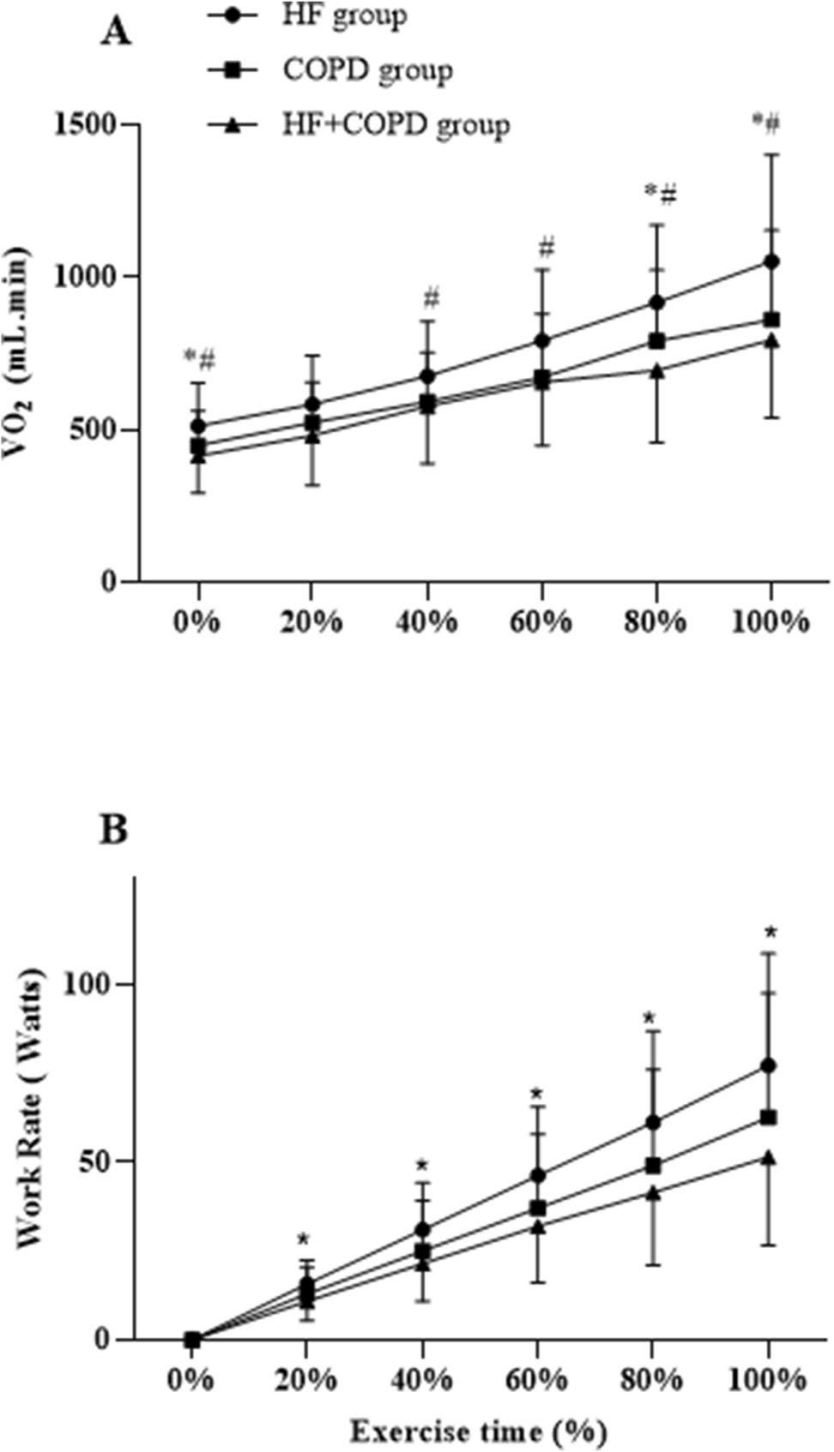
Behavior of ${\dot{\text{V}}}$O_2_ and WR between groups during exercise time. *Significant difference (p < 0.05) in relation to the HF with HF + COPD. ^#^Significant difference (p < 0.05) in relation to the HF with COPD.

The HF group showed better cardiorespiratory fitness compared to the other groups when we evaluated different moments of the incremental exercise (Fig. 3). From rest to peak exercise, the increment in ${\dot{\text{V}}}$O_2_ and WR were greater in the HF group compared to the COPD and HF + COPD groups.

### Relationships between measures of pulmonary function, anthropometric measures, clinical characteristics and exercise responses

**Figure 4:**
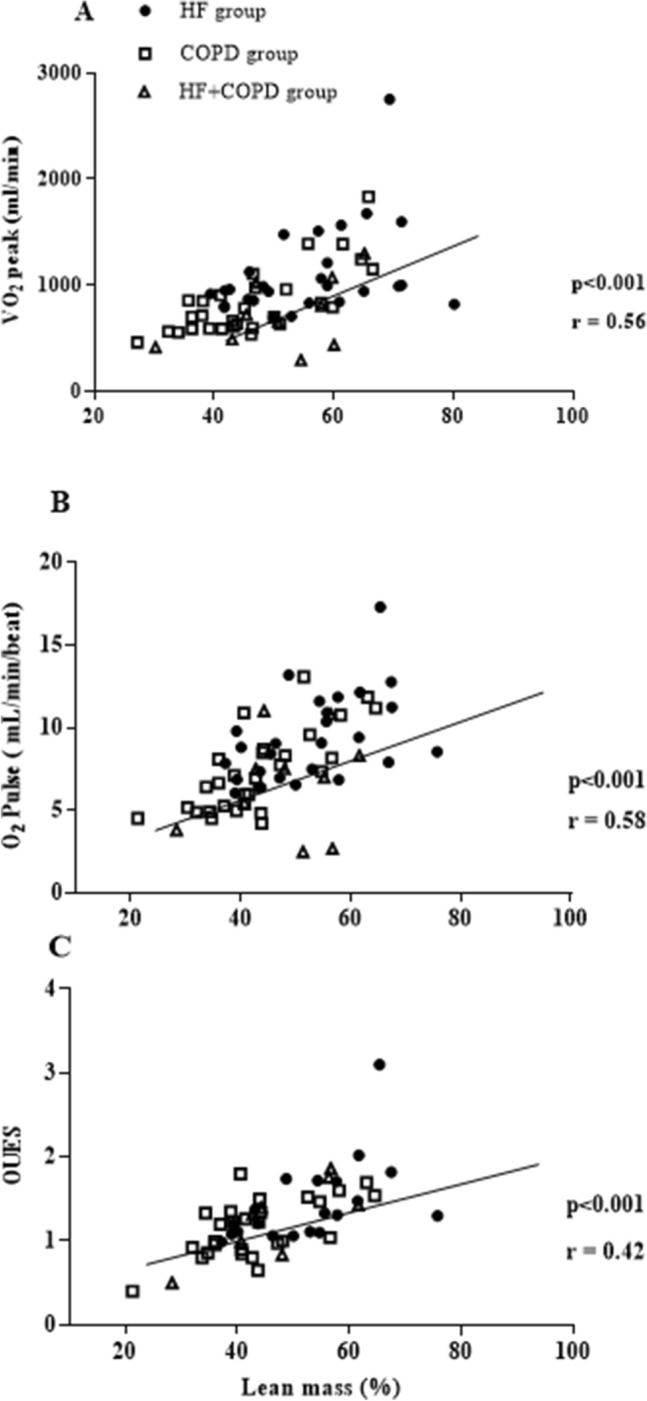
Correlation between body composition and CPET responses; Used Pearson correlation coefficient. (**A**) Relationship between lean mass and oxygen uptake (${\dot{\text{V}}}$O_2_); (**B**) relationship between lean mass and Oxygen pulse; (**C**) relationship between lean mass and oxygen uptake efficiency slope.

**Figure 5:**
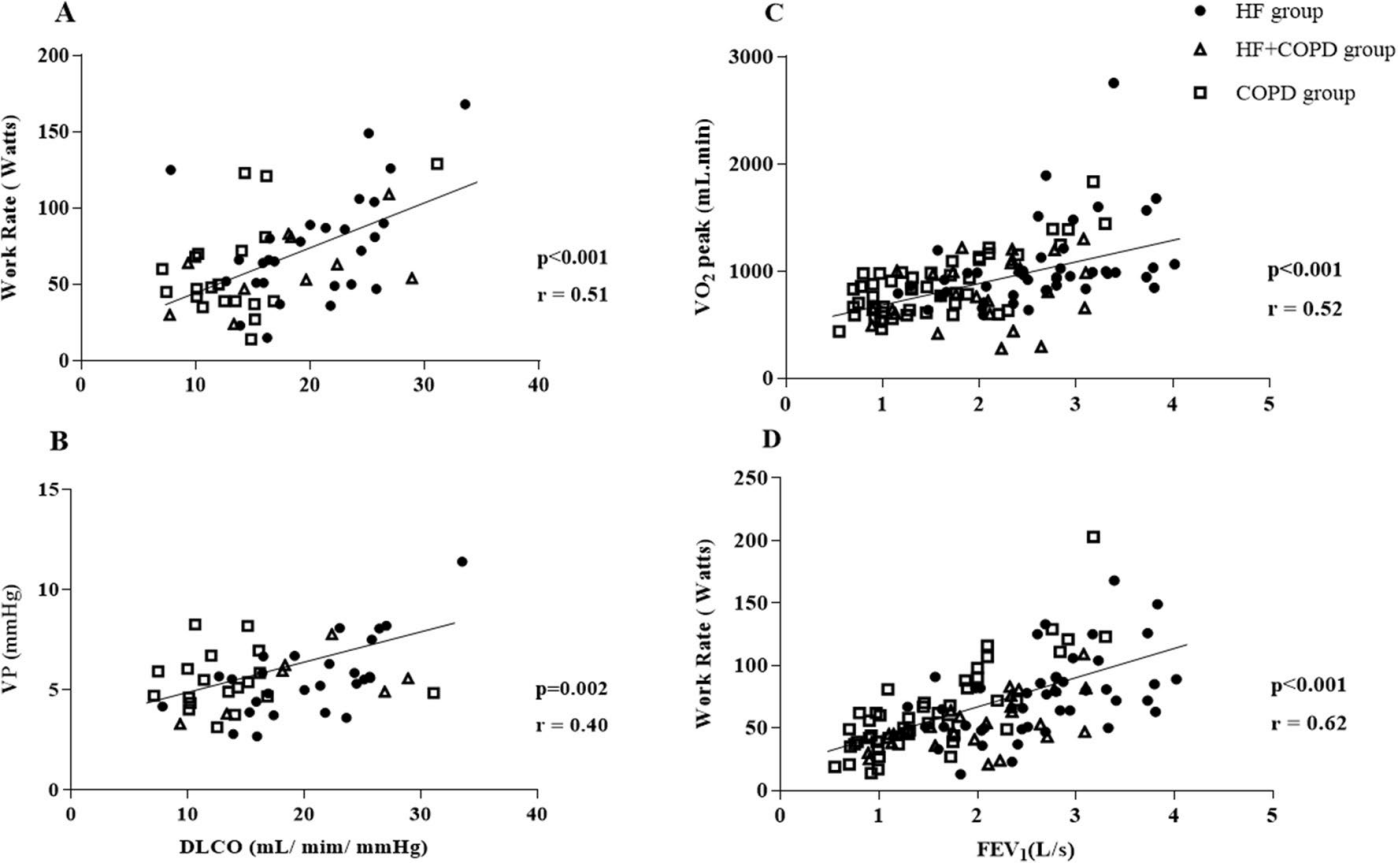
Correlation between lung function and CPET responses; used Pearson correlation coefficient. (**A**) Relationship between diffusion capacity carbon monoxide (DLCO) and work rate; (**B**) relationship between DLCO and ventilatory power; (**C**) relationship between forced expiratory volume in the first second (FEV_1_) and peak oxygen uptake (${\dot{\text{V}}}$O_2_); (**D**) relationship between FEV_1_ and peak work rate. Patients that pefromed DLCO: 58 (HF: 28, COPD: 20, HF + COPD: 10).

We found associations between components of pulmonary function, anthropometric measures, clinical characteristics and CPET variables (Figs. 4, 5). Combining the three groups, significant correlations were observed between lean mass and peak ${\dot{\text{V}}}$O_2_ (r: 0.56 p < 0.001), lean mass and OUES (r: 0.42 p < 0.001), lean mass and peak O_2_ pulse (r: 0.58 p < 0.001), D_LCO_ and WR (r: 0.51 p < 0.001), and D_LCO_ and VP (r: 0.40 p: 0.002). In addition, we found that FEV_1_ was correlated with peak ${\dot{\text{V}}}$O_2_ (r: 0.52; p < 0.001) and peak WR (r: 0.62; p < 0.001).

### Occurrence of cardiopulmonary events in the follow-up period

**Figure 6:**
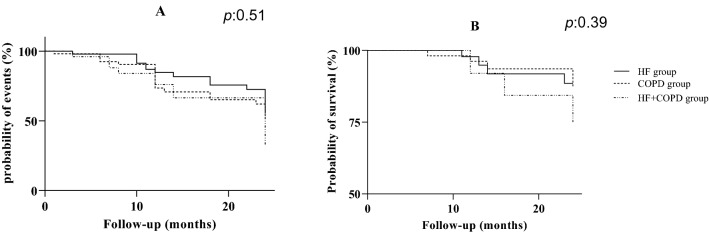
Kaplan–Meier curve. (**A**) Probability of occurrence of cardiopulmonary events; (**B**) probability of survival assessed by occurrence of deaths.

In Table 3, we can see that the occurrence of the events obtained was not different between the groups. The Fig. 6 shows the Kaplan–Meier analysis in both groups. No differences were found between groups in cardiopulmonary outcomes when evaluating disease exacerbations, hospitalizations for cardiopulmonary causes, acute myocardial infarction, stroke, cardiac/pulmonary surgery (Fig. 6A) and survival (Fig. 6B).

**Table 3:** Occurrence of cardiopulmonary events during the follow-up period.

Outcomes	HF n = 46	COPD n = 53	HF + COPD n = 25	*p*
Disease exacerbation, n (%)	16 (34.7)	20 (37.7)	10 (40)	0.85
AMI, n (%)	3 (6.5)	0 (0)	0 (0)	0.07
Stroke, n (%)	3 (6.9)	4 (7.5)	1 (4)	0.97
Hospitalization, n (%)	10 (21.7)	13 (28.2)	7 (15.2)	0.40
Death, n (%)	4 (6.5)	5 (9.4)	5 (16)	0.59
Others, n (%)	9 (19.6)	4 (7.5)	1 (4)	0.07

*AMI* acute myocardial infarction, *Others* gastric or renal or intestinal surgery, decompensation of non-cardiopulmonary diseases.

## Discussion

The main original findings of the present investigation involving patients with HF, COPD and coexisting HF + COPD are as follows: (1) the prevalence of HF + COPD overlap was 20% in the studied patients. (2) Patients with HF + COPD had the greatest impairment with cardiorespiratory fitness, expressed by lower values in key CPET variables; (3) when groups were contrasted at different distinct exercise time points, the HF group has better responses compared to the other two groups, including WR, peak ${\dot{\text{V}}}$O_2_; (4) correlations in the overall group suggest that components of pulmonary function and anthropometric characteristics can influence CPET variables; (5) after the follow-up period of both groups, no differences were found in the occurrence of cardiopulmonary events and deaths.

The aging population is a worldwide phenomenon; due to the health issues associated with aging, a higher proportion of the global population are at risk for chronic disease and diagnoses as well as comorbidity^34^. A coexistance of HF and COPD (overlap syndrome) has been associated with increased morbidity and decreased quality of life as well as a greater use of health resources; the literature indicates a HF + COPD prevalence between 10 and 30%^11,35,36^. Age, sex and anthropometric characteristics have been shown to influence cardiorespiratory fitness^37^. In our study, these factors were different between groups, being that the COPD and HF + COPD groups were older than the HF group (p < 0.05), moreover body composition was different between HF and COPD groups. These findings may indicate an advantage for this group, although the groups are in an age range between 60 and 70 years on average. These differences may explain the slightly more favorable exercise response in the HF group.

Left ventricular EF has been an important survival marker in patients with cardiopulmonary diseases. It has been shown that age has an influence on systolic function, and although differences in EF were not found between HF and HF + COPD, the mean age of the overlap group was higher. Shah et al. assessed 18,398 subjects with reduced EF and found that the mean survival of patients aged 65–69 was 4 years^38,39^. In relation to pulmonary function, no differences were found between the two groups with COPD in spirometric values. Nevertheless, static lung values demonstrated that the COPD group presented with compatible volumes with greater air trapping and a worse D_LCO_. In general, inflammation and structural changes in the airways resulting from COPD increase expiratory flow limitations and worsens with advancing disease severity. The destruction of the lung parenchyma in association with airflow limitation generates air trapping that results in hyperinflation which associated with a reduced IC leads to reduced ventilation-perfusion and limited gas diffusion compromising the gas exchange^40,41^. Hyperinflation in association with changes in alveolar ventilation (VA), minute ventilation and reduction of the pulmonary vascular bed leads to hypoventilation and with the progression of the disease there may be an increase in PaCO_2_ leads to conditions of hypercapnia and hypoxia^41,42^.

It is already well known that patients with HF and COPD have reduced exercise performance, due to impaired ventilatory function and systemic manifestations that affect the muscular and cardiopulmonary system, increasing the limitation to exertion^43,44^. CPET allows a rigorous evaluation of the interaction between respiratory deficiencies caused by diseases and reduced exercise capacity in individuals under physiological stress, being possible to verify which is the main limiting factor to physical exercise^45^. Thus, in the present study, the HF + COPD group had a lower WR when compared to the HF group. The association between advanced age, intrapulmonary conditions, impaired cardiovascular function and loss of muscle strength and endurance in patients with HF + COPD leads to impaired performance during exercise, and intolerance to high workloads^46,47^. Interestingly, no differences were found in the % of WR predicted achieved at peak exercise between groups. This can be explained by the fact that, due to differences in age, height and weight influence the predicted WR. Thus, although it seems that the individuals performed the same effort in the test, the predicted WR was different between the groups, with each group reaching values of % of the predicted WR, proportionally to the predicted value of the WR.

The V̇O_2_ is the main marker of aerobic capacity. In our study, we only found differences in absolute values; no differences were found for the relative values. A combination of factors leads to a reduction in peak ${\dot{\text{V}}}$O_2_ of patients with HF + COPD: ventilatory abnormalities that generate inefficiency in capitation, changes in the heart pump that lead to impaired delivery, and changes in muscle cell composition that contribute to reduced oxygen utilization^48,49^. When we contrasted the groups during the entire exercise time, we can see that the HF group had better performance in the exercise, and a combination of factors such us a younger age group, preserved lung function and most individuals in this sample with mild systolic dysfunction (reflected better physiological adjustments to exercise) may have contributed to these individuals tolerating longer exercise time, greater load increments and consequently higher absolute VO_2_ values.

A comprehensive assessment of several measures obtained from CPET provide for a more comprehensive cardiorespiratory fitness evaluation^50^. The O_2_ pulse is a strong predictor of disease severity and adverse events^51^. Mathematically, the O_2_ pulse is determined by the product of the stroke volume and arteriovenous oxygen difference, and changes in O_2_ pulse during exercise suggested alterations in the stroke volume^52^. In the current study, we observes that the HF group, when compared to the COPD group, have higher O_2_ pulse values. The RPP was significantly lower in the HF + COPD group when compared with the COPD group, indicating poorer cardiac function, as expected in this comorbidity group. The influence of HF in the group with overlap syndrome can increase myocardial oxygen consumption, leading to exhaustion of coronary blood flow reserve and impaired myocardial perfusion^53^. Another important variable that reflects central and peripheral components of cardiac work is CP. Our HF + COPD group presented with worse CP values, this can be explained by the fact that the association of the two diseases leads to reduced cardiac function, which during high-intensity exercise can lead to pulmonary congestion, and that, associated with higher pulmonary arterial pressure and pulmonary vascular resistance, increases ventilation–perfusion incompatibility, producing ventilatory inefficiency and contributing to low CP values^54^.

In recent years, more complex variables derived from CPET have proved to be strong prognostic variables, capable of providing complementary and superior information compared to the isolated use of peak ${\dot{\text{V}}}$O_2_^55^. In our study, no differences were found for the ${\dot{\text{V}}}$_E_/VCO_2_ slope, although all groups demonstrated a mean value above the normal threshold (i.e., > 30), thus observing a worse prognosis for these patients. Arena et al. followed 213 cardiac patients and found that ${\dot{\text{V}}}$_E_/${\dot{\text{V}}}$CO_2_ slope values ≥ 34 associated with ${\dot{\text{V}}}$O_2_ peak ≤ 14 mL kg^–1^ min^−1^ were strong predictors of hospitalization and mortality^56^.

The ${\dot{\text{V}}}$_E_/${\dot{\text{V}}}$CO_2_ intercept is a new parameter, and in patients with lung diseases it increases with the severity of disease^57^. Surprisingly, only the COPD group had higher values of ${\dot{\text{V}}}$_E_/${\dot{\text{V}}}$CO_2_ intercept, suggesting an increased dead space on exercise, something that was not seen in our overlap group. Nevertheless, HF + COPD presented reduced VP in comparison with HF group (p < 0.05). The VP has been studied as a prognostic marker in cardiopulmonary diseases^58,59^. This variable reflects peak cardiac output, alveolar perfusion, peripheral perfusion and the chemo-afferent reflexes of the skeletal muscle, with values below 3.5 mmHg indicating worsening survival^32^.

Lung volumes at rest influence exercise capacity and directly contribute to ventilatory inefficiency during exercise, The Air trapping does not allow all inhaled gas to be exhaled, generating hyperinflation that reduces the inspiratory capacity, influencing the increase in functional residual capacity (FRC), causing these individuals to breathe more quickly during exercise with a tidal volume (VT) reduced, which associated with low lung compliance and ventilatory muscle weakness, make these patients deplete their ventilatory reserve earlier on exercise^60,61^. In this context, greater neural activity is needed to increase the muscle contractions necessary to generate adequate ventilation. This increase in the efferent direction of these muscles can contribute to the development of dyspnea, which when combined with a high respiratory effort due to severe obstruction and hyperinflation, generate greater respiratory muscle impairment^61^.

Pulmonary and systemic cardiocirculatory maladjustments occur in both HF and COPD and in individuals with overlap, these effects are more prominent. Muscle weakness is the most common systemic effect, as it occurs in chronic processes such as what occurs in both diseases^62^. The increase in circulating inflammatory cytokines, such as tumor necrosis factor α, interleukins 6 and 1β, increase oxidative stress and the increase in reactive oxygen species (ROS) associated with tissue hypoxia, provide protein degradation with a consequent reduction in mass muscle and muscle atrophy affecting directly the ventilatory function, which manifest a pronounced intolerance to exercise^63,64^. In the current study, the HF + COPD group had worse perception of symptoms, when compared to the HF group; the perception of dyspnea was higher, and when compared to the COPD group, fatigue values were higher.

Important correlations between clinical variables, body composition and CPET variables were found in this study. It is important to note that lean mass moderately influenced peak ${\dot{\text{V}}}$O_2_, OUES and O_2_ pulse responses in the patients studied. An adequate interaction between the ventilatory, cardiovascular and muscular systems is a determining factor for appropriate oxygen metabolism during incremental exercise. Cardiopulmonary disease initially leads to a compromise of the pulmonary and cardiovascular systems, however, with disease progression, lean mass alterations occur in these populations affecting muscle performance during exercise. These changes occur due factors such as hypoxia, oxidative stress, disuse, nutritional depletion, systemic inflammation and changes in muscle morphology, fiber type distribution and metabolism^65^. Other important findings correlate D_LCO_, and FEV_1_ with peak ${\dot{\text{V}}}$O_2_, WR and VP. Impaired lung function due to air flow limitations, increased intrathoracic pressures, increased intrathoracic blood volume and chronic pulmonary congestion and accumulation of extravascular lung water has a direct effect on the cardiopulmonary response to exercise, leading to increased pulmonary ventilation, ventilatory inefficiency and, consequently, low peak ${\dot{\text{V}}}$O_2_ and WR^11,66^.

Surprisingly, we found no differences between the outcomes assessed in the follow-up of patients in both groups. Although the HF + COPD group has a greater impairment of cardiorespiratory fitness, it is possible to note that the HF and COPD groups also present values of key cardiopulmonary variables for the studied populations compatible with a worse prognosis and high risk of adverse events in the period from 1 to 4 years of follow-up^67^. Despite the association of comorbidities in individuals with cardiopulmonary diseases increases the risk of clinical events and mortality and in our study the comorbidities were different between groups we believe that the advances in the clinical treatment and the optimized pharmacological therapy favored better control of these diseases, thus, mitigating the risk of events^68^. Furthermore, the increasing access to cardiopulmonary rehabilitation programs has favored the reduction of adverse events in patients with cardiopulmonary diseases^69–73^.

### Study limitations

This study has some limitations which are inherent to its nature that consider the screening of patients at 3 ambulatory clinics (pneumology and cardiology) diagnosed with at least one of the diseases and aged over 50 years. As the purpose of the present study was to evaluate the coexistence of one condition in the other, it would be expected that some clinical variables would be different. In this context, it was not possible to pair the groups by disease severity, furthermore, the absence of female individuals in the HF + COPD group, the difference of age between groups and the difference in the body composition can influence the CPET response. However, to mitigate this bias, knowing that some variables could be influenced by age and sex, we performed a linear regression analysis to verify the influence on CPET variables that differed. We verified that age and sex had weak but significant influence on WR (R^2^:0.22 p: 0.000), absolute peak *V̇*O_2_ (R^2^: 0.25 p: 0.000), O_2_ pulse (R^2^: 0.20 p: 0.000) and CP (R^2^: 0.09 p: 0.02).

#### Implications of the study

The study compared the diagnosis of HF + COPD overlap with the diagnosis of isolated diseases. The comparison of the 3 groups so far is unprecedented in the literature and further strengthened the hypothesis that the coexistence of the HF + COPD has more serious implications for exercise capacity. However, the follow-up of these patients, carried out in the study, was essential to clarify the questions about the occurrence of cardiopulmonary outcomes in the three populations, thus clarifying that in a period of up to 24 months of follow-up, no differences were found in the outcomes studied between groups. This suggests that studies with longer follow-up periods can provide better clarification regarding the severity of the HF + COPD overlap and the functional decline that occurs in the studied population. In addition, conducting a study with a monitoring period of 24 months in this population with overlap of HF + COPD difficult to be carried out, standing out the importance of this study.

## Conclusions

In conclusion, the coexistence of HF + COPD induces greater impairment on exercise performance when compared to patients without overlapping diseases, however the overlap of the two diseases did not increase the probability of the occurrence of cardiopulmonary events and deaths when compared to groups with isolated diseases in the period studied. CPET provides important information to guide effective strategies for these patients with the goal of improving exercise performance and functional capacity. Moreover, given our findings related to pulmonary function, body composition and exercise responses, evidenced that the lean mass, FEV_1_ and D_LCO_ influence important responses to exercise.
